# Identifying airborne transmission as the dominant route for the spread of COVID-19

**DOI:** 10.1073/pnas.2009637117

**Published:** 2020-06-11

**Authors:** Renyi Zhang, Yixin Li, Annie L. Zhang, Yuan Wang, Mario J. Molina

**Affiliations:** ^a^Department of Atmospheric Sciences, Texas A&M University, College Station, TX 77843;; ^b^Department of Chemistry, Texas A&M University, College Station, TX 77843;; ^c^Department of Chemistry, College of Natural Sciences, The University of Texas at Austin, Austin, TX 78712;; ^d^Division of Geological and Planetary Sciences, California Institute of Technology, Pasadena, CA 91125;; ^e^Department of Chemistry and Biochemistry, University of California San Diego, La Jolla, CA 92093

**Keywords:** COVID-19, virus, aerosol, public health, pandemic

## Abstract

Various mitigation measures have been implemented to fight the coronavirus disease 2019 (COVID-19) pandemic, including widely adopted social distancing and mandated face covering. However, assessing the effectiveness of those intervention practices hinges on the understanding of virus transmission, which remains uncertain. Here we show that airborne transmission is highly virulent and represents the dominant route to spread the disease. By analyzing the trend and mitigation measures in Wuhan, China, Italy, and New York City, from January 23 to May 9, 2020, we illustrate that the impacts of mitigation measures are discernable from the trends of the pandemic. Our analysis reveals that the difference with and without mandated face covering represents the determinant in shaping the pandemic trends in the three epicenters. This protective measure alone significantly reduced the number of infections, that is, by over 78,000 in Italy from April 6 to May 9 and over 66,000 in New York City from April 17 to May 9. Other mitigation measures, such as social distancing implemented in the United States, are insufficient by themselves in protecting the public. We conclude that wearing of face masks in public corresponds to the most effective means to prevent interhuman transmission, and this inexpensive practice, in conjunction with simultaneous social distancing, quarantine, and contact tracing, represents the most likely fighting opportunity to stop the COVID-19 pandemic. Our work also highlights the fact that sound science is essential in decision-making for the current and future public health pandemics.

## Significance

We have elucidated the transmission pathways of coronavirus disease 2019 (COVID-19) by analyzing the trend and mitigation measures in the three epicenters. Our results show that the airborne transmission route is highly virulent and dominant for the spread of COVID-19. The mitigation measures are discernable from the trends of the pandemic. Our analysis reveals that the difference with and without mandated face covering represents the determinant in shaping the trends of the pandemic. This protective measure significantly reduces the number of infections. Other mitigation measures, such as social distancing implemented in the United States, are insufficient by themselves in protecting the public. Our work also highlights the necessity that sound science is essential in decision-making for the current and future public health pandemics.

The novel coronavirus outbreak, coronavirus disease 2019 (COVID-19), which was declared a pandemic by the World Health Organization (WHO) on March 11, 2020, has infected over 4 million people and caused nearly 300,000 fatalities over 188 countries (1). Intensive effort is ongoing worldwide to establish effective treatments and develop a vaccine for the disease. The novel coronavirus, named as severe acute respiratory syndrome coronavirus 2 (SARS-CoV-2), belongs to the family of the pathogen that is responsible for respiratory illness linked to the 2002–2003 outbreak (SARS-CoV-1) (2). The enveloped virus contains a positive-sense single-stranded RNA genome and a nucleocapsid of helical symmetry of ∼120 nm. There exist several plausible pathways for viruses to be transmitted from person to person. Human atomization of virus-bearing particles occurs from coughing/sneezing and even from normal breathing/talking by an infected person (3–6). These mechanisms of viral shedding produce large droplets and small aerosols (3), which are conventionally delineated at a size of 5 μm to characterize their distinct dispersion efficiencies and residence times in air as well as the deposition patterns along the human respiratory tract (3, 7). Virus transmission occurs via direct (deposited on persons) or indirect (deposited on objects) contact and airborne (droplets and aerosols) routes (3). Large droplets readily settle out of air to cause person/object contamination; in contrast, aerosols are efficiently dispersed in air. While transmission via direct or indirect contact occurs in a short range, airborne transmission via aerosols can occur over an extended distance and time. Inhaled virus-bearing aerosols deposit directly along the human respiratory tract.

Previous experimental and observational studies on interhuman transmission have indicated a significant role of aerosols in the transmission of many respiratory viruses, including influenza virus, SARS-CoV-1, and Middle East Respiratory Syndrome coronavirus (MERS-CoV) (8–11). For example, airborne coronavirus MERS-CoV exhibited strong capability of surviving, with about 64% of microorganisms remaining infectious 60 min after atomization at 25 °C and 79% relative humidity (RH) (9). On the other hand, rapid virus decay occurred, with only 5% survival over a 60-min procedure at 38 °C and 24% RH, indicative of inactivation. Recent experimental studies have examined the stability of SARS-CoV-2, showing that the virus remains infectious in aerosols for hours (12) and on surfaces up to days (12, 13).

Several parameters likely influence the microorganism survival and delivery in air, including temperature, humidity, microbial resistance to external physical and biological stresses, and solar ultraviolet (UV) radiation (7). Transmission and infectivity of airborne viruses are also dependent on the size and number concentration of inhaled aerosols, which regulate the amount (dose) and pattern for respiratory deposition. With typical nasal breathing (i.e., at a velocity of ∼1 m⋅s^−1^) (4), inhalation of airborne viruses leads to direct and continuous deposition into the human respiratory tract. In particular, fine aerosols (i.e., particulate matter smaller than 2.5 μm, or PM_2.5_) penetrate deeply into the respiratory tract and even reach other vital organs (14, 15). In addition, viral shedding is dependent on the stages of infection and varies between symptomatic and asymptomatic carriers. A recent finding (16) showed that the highest viral load in the upper respiratory tract occurs at the symptom onset, suggesting the peak of infectiousness on or before the symptom onset and substantial asymptomatic transmission for SARS-CoV-2.

The COVID-19 outbreak is significantly more pronounced than that of the 2002/2003 SARS, and the disease continues to spread at an alarming rate worldwide, despite extreme measures taken by many countries to constrain the pandemic (1). The enormous scope and magnitude of the COVID-19 outbreak reflect not only a highly contagious nature but also exceedingly efficient transmission for SARS-CoV-2. Currently, the mechanisms to spread the virus remain uncertain (17), particularly considering the relative contribution of the contact vs. airborne transmission routes to this global pandemic. Available epidemiological (1) and experimental (12, 18) evidence, however, implicates airborne transmission of SARS-CoV-2 via aerosols as a potential route for the spreading of the disease.

## Distinct Pandemic Trends in the Three Epicenters

**Fig. 1.**
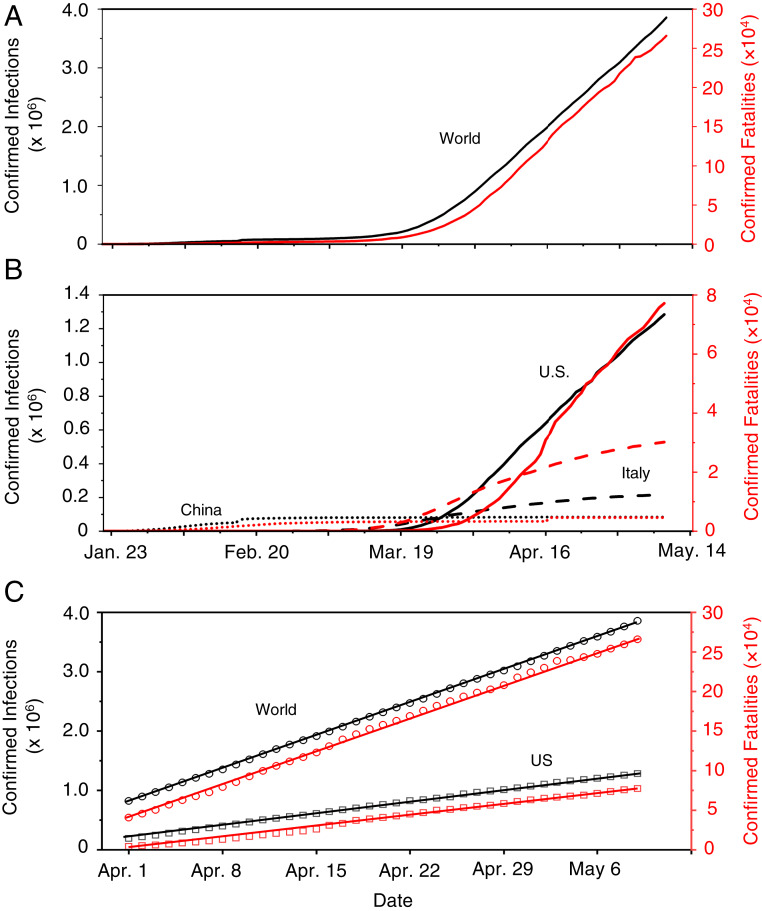
Distinct global trends of the COVID-19 pandemic. (*A*) Confirmed infections and fatalities worldwide. (*B*) Comparison of the confirmed infections and fatalities between China, Italy, and United States. (*C*) Linear regression of the confirmed infections and fatalities worldwide and in United States from April 1 to May 9, 2020; the linear regression is, respectively, *y* = 79,398*x* + 810,167 (*R*^2^ = 0.999) for infections and *y* = 6,075*x* + 39,409 (*R*^2^ = 0.998) for fatalities worldwide and *y* = 28,971*x* + 201,187 (*R*^2^ = 0.999) for infections and *y* = 2,059*x* + 243 (*R*^2^ = 0.995) for fatalities in the United States. The left axis and black color correspond to the numbers of confirmed infections, and the right axis and red color represent the confirmed fatalities.

To gain insight into the mechanism of the virus transmission routes and assess the effectiveness of mitigation measures, we analyzed the trend of the pandemic worldwide from January 23 to May 9, 2020 (Fig. 1). The COVID-19 outbreak initially emerged during December 2019 in Wuhan, China (1). The numbers of confirmed infections and fatalities in China dominated the global trend during January and February 2020 (Fig. 1*A*), but the increases in the newly confirmed cases and fatalities in China have exhibited sharp declines since February (Fig. 1*B*). In contrast to the curve flattening in China, those numbers in other countries have increased sharply since the beginning of March. The epicenter shifted from Wuhan to Italy in early March and to New York City (NYC) in early April. By April 30, the numbers of confirmed COVID-19 cases and deaths, respectively, reached over 200,000 and 27,000 in Italy and over 1,000,000 and 52,000 in the United States, compared to about 84,000 and 4,600 in China (Fig. 1*B*). Notably, the curves in Italy exhibit a slowing trend since mid-April, while the numbers in the world and the United States continue to increase. Remarkably, the recent trends in the numbers of infections and fatalities in the world and in the United States exhibit striking linearity since the beginning of April (Fig. 1*C*).

We interpreted the differences in the pandemic trends by considering the mitigation measures implemented worldwide. The curve flattening in China can be attributed to extensive testing, quarantine, and contact tracing; other aggressive measures implemented in China include lockdown of all cities and rural areas in the whole country, isolation of residents having close contact with infected people, and mandated wearing of face masks in public. However, the effectiveness of those mitigation measures has yet to be rigorously evaluated. Differentiation of the effects of those mitigation measures in China is challenging (19), since the implementation occurred almost simultaneously in January 2020. While similar quarantine, isolation, and city lockdown measures were also implemented on March 9 in Italy after the country became the second epicenter, the curve of infections has yet to show complete flattening. In the United States, guidelines for social distancing, quarantine, and isolation were issued by the federal government on March 16, and stay-at-home orders were implemented by many state and local governments starting, for example, on March 19 and April 3 and on March 22 in NYC. The social distancing measures implemented in the United States include staying at least 6 feet (∼2 m) away from other people, no gathering in groups, staying out of crowded places, and avoiding mass gatherings (20). Obviously, the continuous rise in the US infected numbers casts doubt on the effectiveness of those preventive measures alone (Fig. 1 *B* and *C*).

In contrast to China, wearing of face masks was not mandated and was unpopular in most of the western world during the early outbreak of the pandemic. Advice on the use of face masks was not issued until April 6, 2020 by the WHO (1), claiming that it is important only to prevent infected persons from viral transmission by filtering out droplets but that it is unimportant to prevent uninfected persons from breathing virus-bearing aerosols. The regions heavily plagued by COVID-19 in northern Italy, such as Lombard, ordered face covering in public starting on April 6, and the Italian authorities required nationwide mandatory use of face masks on May 4. All New Yorkers were mandated to use face covering in public starting on April 17, when social distancing was not possible. With measures implemented in the United States seemingly comparable to those in China, social distancing, quarantine, and isolation exhibited little impact on stopping the spreading of the disease in the United States, as reflected by the linearity from April 1 to May 9 (Fig. 1*C*). It is possible, however, that these measures likely alter the slope of the infection curve, that is, by reducing the rate of infections during the early stage of the pandemic (Fig. 1). Notably, the recommended physical separation for social distancing is beneficial to prevent direct contact transmission but is insufficient (without face masks) to protect inhalation of virus-bearing aerosols (or even small droplets at intermediate proximity), owing to rapid air mixing (7).

## Understanding the Impacts of Face Covering

**Fig. 2.**
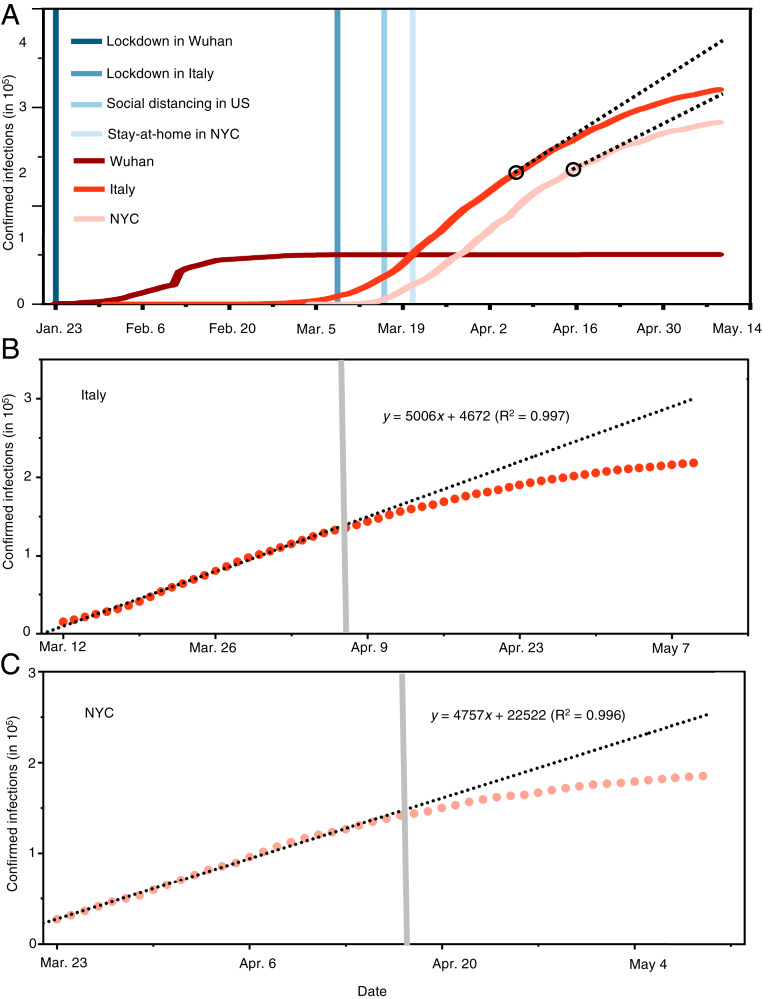
The evolving epicenter from Wuhan, to Italy, to NYC. (*A*) Comparison of the trends and mitigation measures between Wuhan, Italy, and NYC in 2020. The vertical lines mark the date for implementing mitigation measures. The two black circles label the dates when face covering was implemented: April 6 in northern Italy and April 17 in NYC. The black dashed lines represent the projection without face covering based on linear regression of 26-d data prior to implementing this measure. (*B*) Linear regression of the number of confirmed infections for 26-d data prior to implementing face covering in Italy. The shaded vertical line denotes the date when face covering was implemented on April 6 in northern Italy. (*C*) Linear regression of the number of confirmed infections for 26-d data prior to implementing face covering in NYC. The shaded vertical line denotes the date when face covering was implemented on April 17 in NYC. In *B* and *C*, the circles are reported values, and the dotted line represents fitting and projection of the confirmed infections before and after face-covering, respectively.

Compared to the simultaneous implementation of measures in China, intervention measures were successively implemented in the western world (Fig. 2*A*), providing an opportunity for assessing their relative effectiveness. We quantified the effects of face covering by projecting the number of infections based on the data prior to implementing the use of face masks in Italy on April 6 and NYC on April 17 (Fig. 2*A*; see *Methods*). Such projections are reasonable considering the excellent linear correlation for the data prior to the onset of mandated face covering (Fig. 2 *B* and *C* and *SI Appendix*, Fig. S1). Our analysis indicates that face covering reduced the number of infections by over 78,000 in Italy from April 6 to May 9 and by over 66,000 in NYC from April 17 to May 9. In addition, varying the correlation from 15 d to 30 d prior to the onset of the implementation reveals little difference in the projection for both places, because of the high correlation coefficients (*SI Appendix*, Fig. S1). Notably, the trends of the infection curves in Italy and NYC contrast to those in the world and in the United States (Fig. 1*C*), which show little deviation from the linearity due to the nonimplementation of face-covering measures globally and nationally, respectively. The inability of social distancing, quarantine, and isolation alone to curb the spread of COVID-19 is also evident from the linearity of the infection curve prior to the onset of the face-covering rule in Italy on April 6 and in NYC on April 17 (Fig. 2 *B* and *C*). Hence, the difference made by implementing face covering significantly shapes the pandemic trends worldwide.

**Fig. 3.**
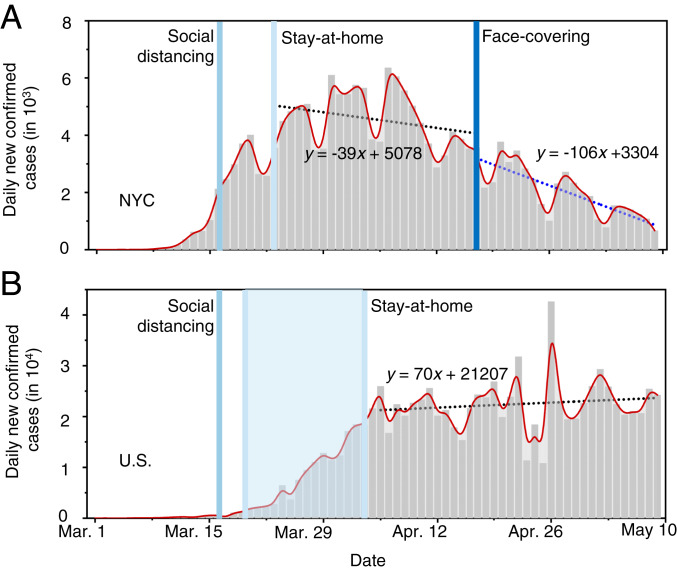
Contrasting the trends of new infections between NYC and the United States. Daily new confirmed infections in (*A*) NYC and (*B*) the United States. The dotted lines represent linear fitting to the data between April 17 and May 9 in NYC and between April 4 and May 9 in the United States. In *B*, the number in NYC was subtracted from that in the United States. The vertical lines label the dates for social distancing, stay-at-home orders, and mandated face-covering.

We further compared the numbers of daily new cases between NYC and the United States (excluding the data in NYC) from March 1 to May 9 (Fig. 3). The daily numbers of newly confirmed infections in NYC and the United States show a sharp increase in late March and early April. There exists a slower increase in the number after implementation of the stay-at-home order (about 14 d in New York and shortly after April 3 in the United States), which is attributable to the impacts of this measure. After April 3, the only difference in the regulatory measures between NYC and the United States lies in face covering on April 17 in NYC. We applied linear regression to the data between April 17 and May 9 in NYC and between April 5 and May 9 in the United States. While the daily numbers of newly confirmed infections fluctuate considerably, the slope of the regression unambiguously reflects the trend in both data. The daily new infection in NYC decreases with a slope of 106 cases per day after April 17, corresponding to a decreasing rate of ∼3% per day (relative to April 17). For comparison, the daily new infections in the United States (excluding NYC) increase, with a slope of 70 cases per day after April 4, corresponding to an increasing rate of ∼0.3% per day (relative to April 5). Hence, the decreasing rate in the daily new infections in NYC with mandated face covering is in sharp contrast to that in the United States with only social-distancing and stay-at-home measures, further confirming the importance of face covering in intervening the virus transmission.

## Dominant Airborne Transmission

**Fig. 4.**
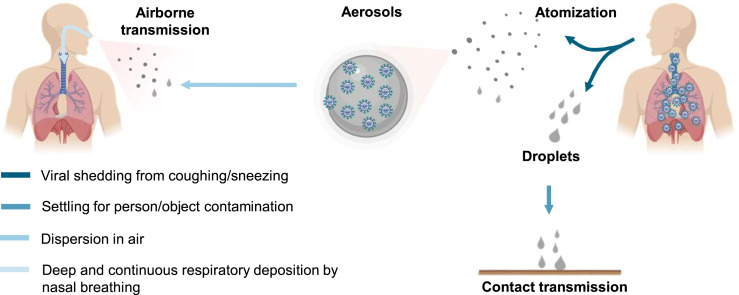
Transmission of COVID-19. Human atomization of viruses arises from coughing or sneezing of an infected person, producing virus-containing droplets (>5 μm) and aerosols (<5 μm). Virus transmission from person to person occurs through direct/indirect contact and airborne aerosol/droplet routes. Large droplets mainly settle out of air to cause person/object contamination, while aerosols are efficiently dispersed in air. Direct and airborne transmissions occur in short range and extended distance/time, respectively. Inhaled airborne viruses deposit directly into the human respiration tract.

We further elucidated the contribution of airborne transmission to the COVID-19 outbreak by comparing the trends and mitigation measures during the pandemic worldwide and by considering the virus transmission routes (Fig. 4). Face covering prevents both airborne transmission by blocking atomization and inhalation of virus-bearing aerosols and contact transmission by blocking viral shedding of droplets. On the other hand, social distancing, quarantine, and isolation, in conjunction with hand sanitizing, minimize contact (direct and indirect) transmission but do not protect against airborne transmission. With social distancing, quarantine, and isolation in place worldwide and in the United States since the beginning of April, airborne transmission represents the only viable route for spreading the disease, when mandated face covering is not implemented. Similarly, airborne transmission also contributes dominantly to the linear increase in the infection prior to the onset of mandated face covering in Italy and NYC (Fig. 2 *B* and *C* and *SI Appendix*, Fig. S1). Hence, the unique function of face covering to block atomization and inhalation of virus-bearing aerosols accounts for the significantly reduced infections in China, Italy, and NYC (Figs. 1–3), indicating that airborne transmission of COVID-19 represents the dominant route for infection.

Recent measurements identified SARS-Cov-2 RNA on aerosols in Wuhan’s hospitals (18) and outdoor in northern Italy (21), unraveling the likelihood of indoor and outdoor airborne transmission. Within an enclosed environment, virus-bearing aerosols from human atomization are readily accumulated, and elevated levels of airborne viruses facilitate transmission from person to person. Transmission of airborne viruses in open air is subject to dilution, although virus accumulation still occurs due to stagnation under polluted urban conditions (7, 22). Removal of virus-bearing particles from human atomization via deposition is strongly size dependent, with the settling velocities ranging from 2.8 × 10^−5^ m⋅s^−1^ to 1.4 × 10^−3^ m⋅s^−1^ for the sizes of 1 and 10 μm, respectively (7). For comparison, typical wind velocity is about 1 m⋅s^−1^ to 3 m⋅s^−1^ indoors (23) and is ∼1 m⋅s^−1^ horizontally and 0.1 m⋅s^−1^ vertically in stable air (7, 22). Under those indoor and outdoor conditions, the residence time of virus-bearing aerosols reaches hours, due to air mixing (7).

We also examined ambient conditions relevant to the outbreaks in Wuhan, Italy, and NYC. The initial outbreak of COVID-19 in Wuhan coincided with the winter haze season in China (7, 22), during which high levels of PM_2.5_ were prevalent in air (*SI Appendix*, Figs. S2 and S3). On the other hand, the daily average PM_2.5_ concentrations were much lower during the outbreaks in Rome, Italy, and in NYC (*SI Appendix*, Fig. S2). The airborne transmission pathways (i.e., indoor or outdoor) as well as the effects of ambient PM_2.5_ levels on virus transmission may be variable among urban cities. For example, the winter haze conditions in China likely exacerbated outdoor virus spreading (24, 25), because of low UV radiation, air stagnation (lacking ventilation on the city scale), and low temperature (7, 22). Also, there may exist a synergetic effect of simultaneous exposure to the virus and PM_2.5_ to enhance the infectivity, severity, and fatalities of the disease (14, 26). In addition, nascent virus-bearing aerosols produced from human atomization likely undergo transformation in air, including coagulation with ambient preexisting PM and/or growth on a time scale of a few hours in typical urban air (27–29). Such transformation, as recently documented on coarse PM in Italy (21), may mitigate virus inactivation (9, 12), by providing a medium to preserve its biological properties and elongating its lifetimes. However, key questions remain concerning transformation and transmission of virus-bearing aerosols from human atomization in air. Specifically, what are the impacts of transformation of human-atomized aerosols on viral surviving and infectivity in air?

While the humidity effect on viral surviving is uncertain (3, 9), the conditions during the outbreaks in Wuhan, Rome, and NYC correspond to high RH yet low absolute humidity because of low temperature (*SI Appendix*, Fig. S3). Early experimental work (9) showed remarkable survival for the analogous coronavirus MERS-CoV at the RH level characteristic of the COVID-19 outbreaks in Wuhan, Rome, and NYC. For comparison, indoor temperature and RH typically range from 21 °C to 27 °C and 20 to 70%, respectively (23).

Of particular importance are the considerations that render airborne SARS-CoV-2 the most efficient among all transmission routes. Even with normal nasal breathing, inhalation of virus-bearing aerosols results in deep and continuous deposition into the human respiratory tract, and this transmission route typically requires a low dose (8). Also, airborne viruses have great mobility and sufficiently long surviving time for dispersion (9, 12), and residents situated in densely populated environments are highly vulnerable. In addition, nascent micrometer-size aerosols produced from coughing/sneezing of infected people have the potential of containing many viruses, particularly for asymptomatic carriers (16).

Future research is critically needed to assess the transmission, transformation, and dispersion of virus-bearing aerosols from human atomization under different environmental conditions, as well as the related impacts on virus infectivity. It is equally important to understand human atomization of airborne viruses: What are the number and size distributions of nascent aerosols as well as the viral load per particle from coughing/sneezing? It is also imperative to evaluate human inhalation of airborne viruses: How are aerosols deposited along the respiratory tract, and what is the minimum dose of airborne viruses required for infection? It is also important to evaluate the performance of face masks to quantify the efficiency to filtrate airborne viruses relevant to human atomization and inhalation. Elucidation of these mechanisms requires an interdisciplinary effort.

## A Policy Perspective

The governments’ responses to the COVID pandemic have so far differed significantly worldwide. Swift actions to the initial outbreak were undertaken in China, as reflected by nearly simultaneous implementation of various aggressive mitigation measures. On the other hand, the response to the pandemic was generally slow in the western world, and implementation of the intervention measures occurred only consecutively. Clearly, the responsiveness of the mitigation measures governed the evolution, scope, and magnitude of the pandemic globally (Figs. 1 and 2).

**Fig. 5.**
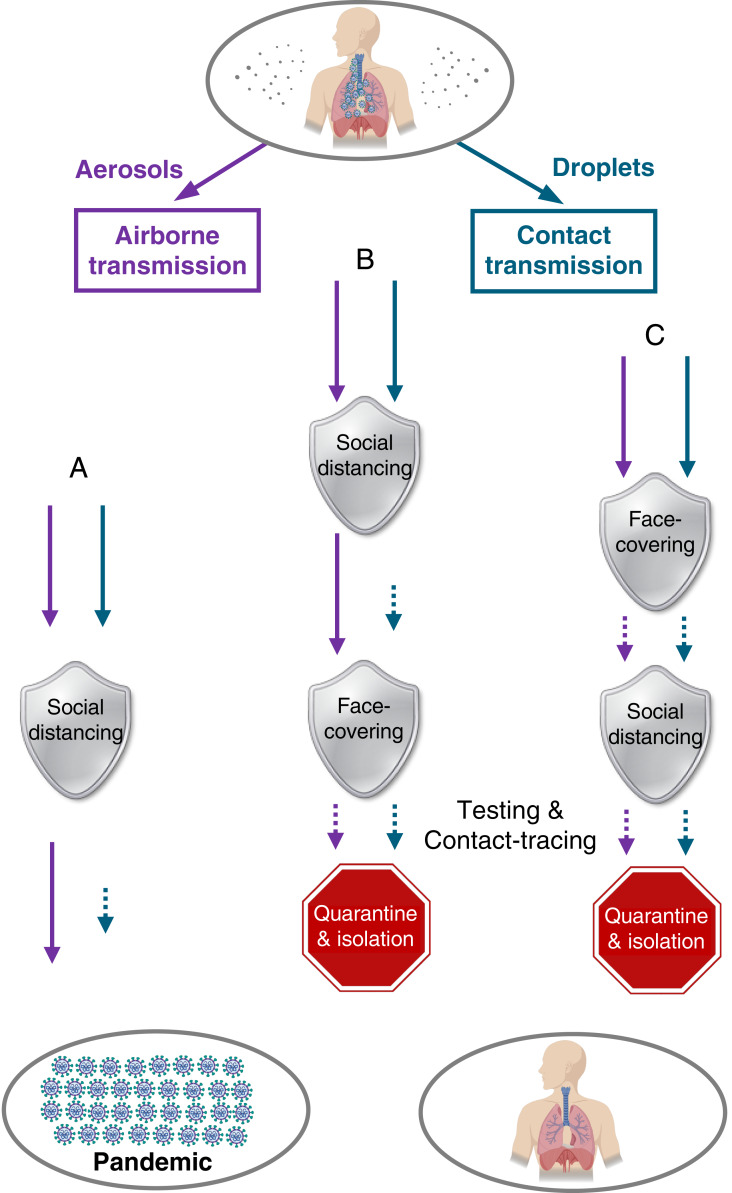
Mitigation paradigm. Scenarios of virus transmission under the distancing/quarantine/isolation measure only (path a), the measures with distancing/quarantine/isolation followed by face covering (path b), and the measures with simultaneous face covering and distancing/quarantine/isolation (path c). The short-dashed arrows label possible remnants of virus transmission due to circumstances when the measure is not possible or disobeyed and/or imperfection of the measure.

Curbing the COVID-19 relies not only on decisive and sweeping actions but also, critically, on the scientific understanding of the virus transmission routes, which determines the effectiveness of the mitigation measures (Fig. 5). In the United States, social distancing and stay-at-home measures, in conjunction with hand sanitizing (Fig. 5, path a), were implemented during the early stage of the pandemic (March 16) (20). These measures minimized short-range contact transmission but did not prevent long-range airborne transmission, responsible for the inefficient containing of the pandemic in the United States (Figs. 1 and 3). Mandated face covering, such as those implemented in China, Italy, and NYC, effectively prevented airborne transmission by blocking atomization and inhalation of virus-bearing aerosols and contact transmission by blocking viral shedding of droplets. While the combined face-covering and social distancing measures offered dual protection against the virus transmission routes, the timing and sequence in implementing the measures also exhibited distinct outcomes during the pandemic. For example, social distancing measures, including city lockdown and stay-at-home orders, were implemented well before face covering was mandated in Italy and NYC (Fig. 5, path b), and this sequence left an extended window (28 d in Italy and 32 d in NYC) for largely uninterrupted airborne transmission to spread the disease (Figs. 2 and 3). The simultaneous implementation of face covering and social distancing (Fig. 5, path c), such as that undertaken in China, was most optimal, and this configuration, in conjunction with extensive testing and contact tracing, was responsible for the curve flattening in China (Fig. 1). Also, there likely existed remnants of virus transmission after the implementation of regulatory measures, because of circumstances when the measures were not practical or were disobeyed and/or imperfection of the measures. Such limitations, which have been emphasized by the WHO (1), spurred on controversial views on the validity of wearing face masks to prevent the virus transmission during the pandemic (30). However, it is implausible that the limitations of mitigation measures alone contributed dominantly to the global pandemic trend, as exemplified by the success in China. Our work suggests that the failure in containing the propagation of COVID-19 pandemic worldwide is largely attributed to the unrecognized importance of airborne virus transmission (1, 20).

## Conclusions

The inadequate knowledge on virus transmission has inevitably hindered development of effective mitigation policies and resulted in unstoppable propagation of the COVID-19 pandemic (Figs. 1–3). In this work, we show that airborne transmission, particularly via nascent aerosols from human atomization, is highly virulent and represents the dominant route for the transmission of this disease. However, the importance of airborne transmission has not been considered in establishment of mitigation measures by government authorities (1, 20). Specifically, while the WHO and the US Centers for Disease Control and Prevention (CDC) have emphasized the prevention of contact transmission, both WHO and CDC have largely ignored the importance of the airborne transmission route (1, 20). The current mitigation measures, such as social distancing, quarantine, and isolation implemented in the United States, are insufficient by themselves in protecting the public. Our analysis reveals that the difference with and without mandated face covering represents the determinant in shaping the trends of the pandemic worldwide. We conclude that wearing of face masks in public corresponds to the most effective means to prevent interhuman transmission, and this inexpensive practice, in conjunction with extensive testing, quarantine, and contact tracking, poses the most probable fighting opportunity to stop the COVID-19 pandemic, prior to the development of a vaccine. It is also important to emphasize that sound science should be effectively communicated to policy makers and should constitute the prime foundation in decision-making amid this pandemic. Implementing policies without a scientific basis could lead to catastrophic consequences, particularly in light of attempts to reopen the economy in many countries. Clearly, integration between science and policy is crucial to formulation of effective emergency responses by policy makers and preparedness by the public for the current and future public health pandemics.

## Methods

Projection of the pandemic trend without implementing face covering in Italy and NYC was performed first by establishing the linear correlation between the infection number and date. We considered the data for both 15 and 30 d prior to the onset of face covering (*SI Appendix*, Fig. S1). The slope and the reported infection number were used for the projections. The avoided infection number due the face covering was determined from the difference between the projected and reported values on May 9, 2020.

The data for accumulative confirmed infections and fatalities in Wuhan, Italy, and NYC were taken from the reports by Wuhan Municipal Health Commission (http://wjw.wuhan.gov.cn/), European CDC (https://www.ecdc.europa.eu/en), and NYC government (https://www1.nyc.gov/site/doh/covid/covid-19-data.page), respectively. The data of accumulative confirmed infections and fatalities worldwide were taken from WHO COVID-19 situation report (https://www.who.int/emergencies/diseases/novel-coronavirus-2019/situation-reports) (1), and the numbers in China, Italy, and United States were from taken from European CDC.

Ground-based measurements of PM_2.5_ and RH in Wuhan were taken from the China National Environmental Monitoring Centre (http://beijingair.sinaapp.com/). The PM_2.5_ data in NYC were taken from US Environmental Protection Agency (https://www.epa.gov/outdoor-air-quality-data). The PM_2.5_ data in Rome were taken were from Centro Regionale della Qualità dell’aria (http://www.arpalazio.net/main/aria/). The RH data in Rome and NYC were taken from the 6-hourly interim reanalysis of the European Centre for Medium-range Weather Forecasts (https://www.ecmwf.int/en/forecasts/datasets/reanalysis-datasets/era5).

We used spaceborne measurements of aerosol optical depth (AOD) to characterize the regional aerosol pollution during the COVID-19 outbreak (January 23 to February 10, 2020) in China. The green band AODs at 0.55 μm are available from Terra and Aqua combined Moderate Resolution Imaging Spectroradiometer Version 6 Multiangle Implementation of Atmospheric Correction (https://lpdaac.usgs.gov/products/mcd19a2v006/). The Level-2 product has daily global coverage with 1-km pixel resolution. The AOD retrieval is only available for the clear sky.

### Data Availability.

All data relevant to this research are available in the main text and *SI Appendix*.

## Supplementary Material

Supplementary File
