# Supplementary material for: Identifying airborne transmission as the dominant route for the spread of COVID-19
Source: Proc Natl Acad Sci U S A. 2020 Jun 11;117(26):14857–63. doi: 10.1073/pnas.2009637117 (PMC7334447; doi:10.1073/pnas.2009637117)
Supplement: Supplementary File [file pnas.2009637117.sapp.pdf]

PNAS
www.pnas.org

Supporting Information for:

Identifying airborne transmission as the dominant route for the spread of COVID-19

Renyi Zhang[a,b,1], Yixin Li[b], Annie L. Zhang[c], Yuan Wang[e] & Mario J. Molina[f,1]

[1]To whom correspondence may be addressed. Email: mjmolina@ucsd.edu and renyi-zhang@tamu.edu

This PDF file includes:

Figs. S1 to S3 (Figs. S1 through S3 are referenced in the main manuscript)

www.pnas.org/cgi/doi/10.1073/pnas.2009637117

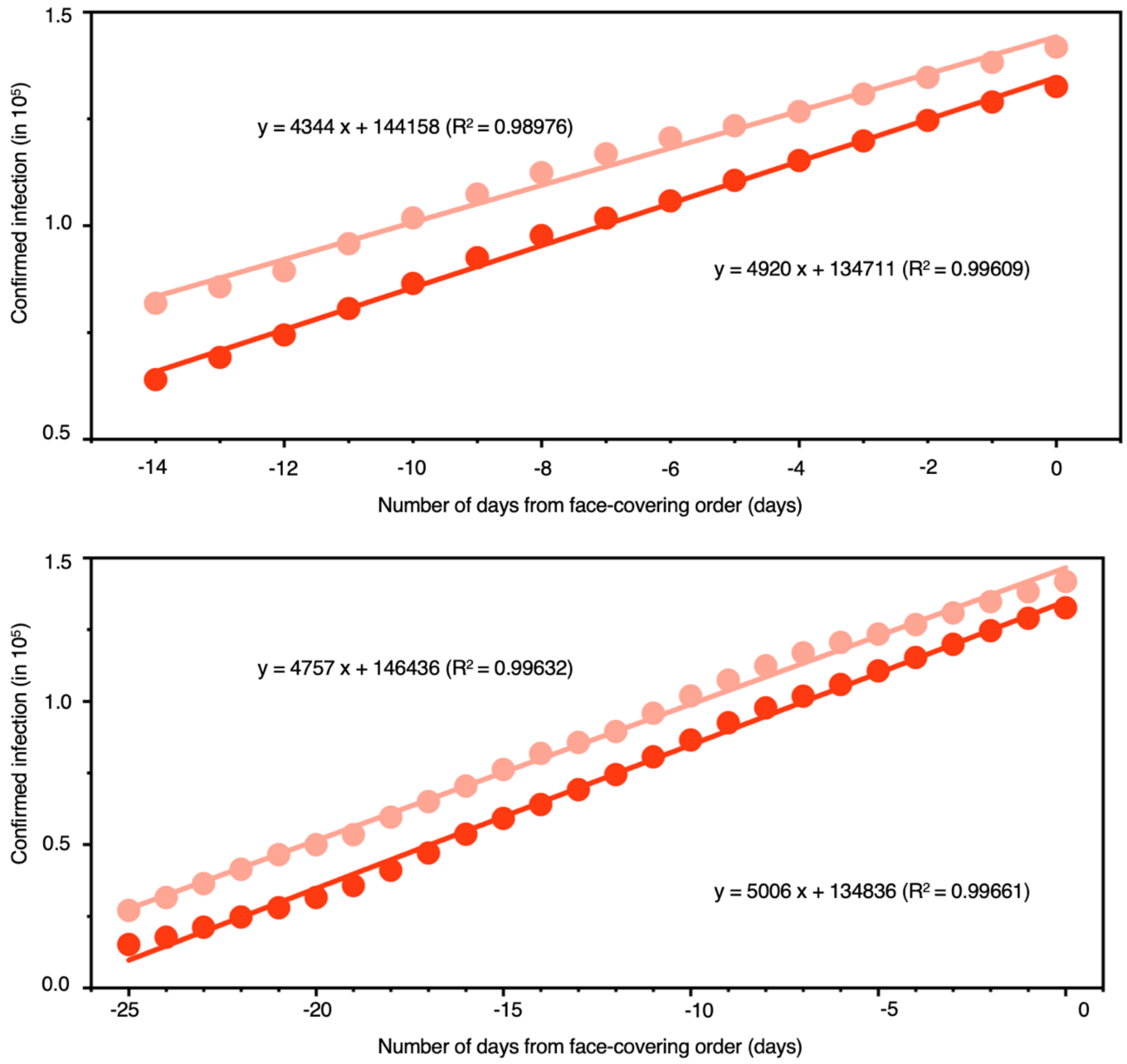

Fig. S1. Linear correlation of the number of confirmed infections for using 15-day (A) and 26-day (B) data prior to implementing face-covering in Italy and NYC.

www.pnas.org/cgi/doi/10.1073/pnas.2009637117

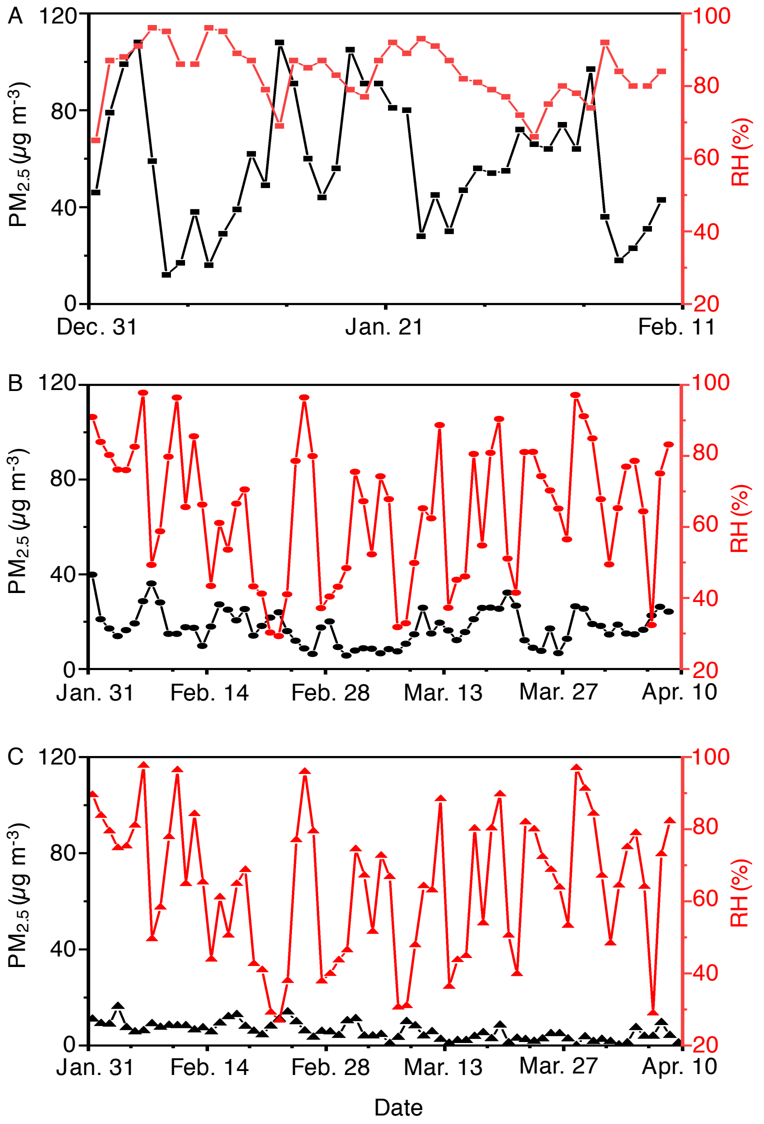

Fig. S2. RH and $PM_{2.5}$ amid the COVID-19 outbreaks in Wuhan (A), Rome (B), and NYC (C).

www.pnas.org/cgi/doi/10.1073/pnas.2009637117

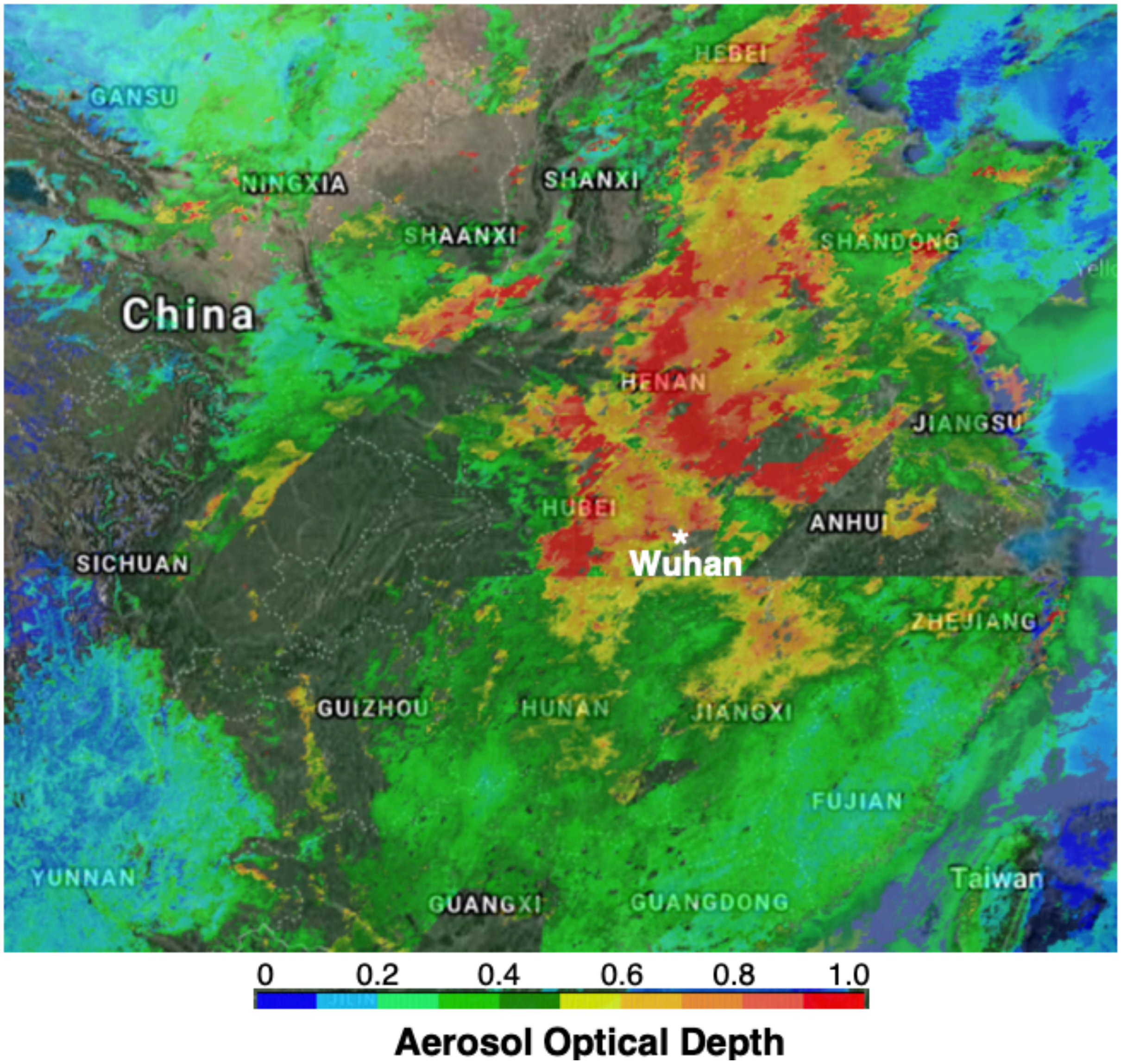

Fig. S3. Mingling of coronavirus COVID-19 with regional haze in Wuhan, China**.** Aerosol optical depth over Eastern China from the Tropospheric Monitoring Instrument (TROPOMI). The data are averaged over January 10 to February 10, 2020. The red (blue) color indicates high (low) aerosol concentration. The city of Wuhan is marked by the white asterisk.
